# Extracellular Vesicles: Decoding a New Language for Cellular Communication in Early Embryonic Development

**DOI:** 10.3389/fcell.2018.00094

**Published:** 2018-08-28

**Authors:** Lilian Cruz, Jenny A. A. Romero, Rebeca P. Iglesia, Marilene H. Lopes

**Affiliations:** Department of Cell and Developmental Biology, Institute of Biomedical Sciences, University of São Paulo, São Paulo, Brazil

**Keywords:** embryonic stem cells, extracellular vesicles, development, miRNAs, morphogens

## Abstract

The blastocyst inner cell mass (ICM) that gives rise to a whole embryo *in vivo* can be derived and cultured *in vitro* as embryonic stem cells (ESCs), which retain full developmental potential. ICM cells receive, from diverse sources, complex molecular and spatiotemporal signals that orchestrate the finely-tuned processes associated with embryogenesis. Those instructions come, continuously, from themselves and from surrounding cells, such as those present in the trophectoderm and primitive endoderm (PrE). A key component of the ICM niche are the extracellular vesicles (EVs), produced by distinct cell types, that carry and transfer key molecules that regulate target cells and modulate cell renewal or cell fate. A growing number of studies have demonstrated the extracellular circulation of morphogens, a group of classical regulators of embryo development, are carried by EVs. miRNAs are also an important cargo of the EVs that have been implicated in tissue morphogenesis and have gained special attention due to their ability to regulate protein expression through post-transcriptional modulation, thereby influencing cell phenotype. This review explores the emerging evidence supporting the role of EVs as an additional mode of intercellular communication in early embryonic and ESCs differentiation.

## Introduction

**Figure 1:**
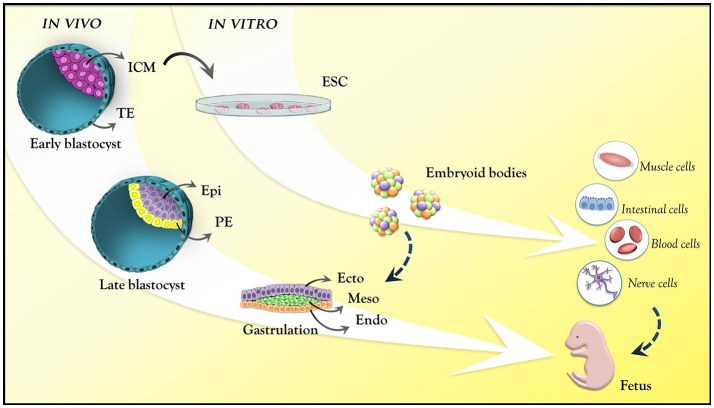
Mouse embryo development at pre- and post-implantation stages. Pre-implantation stage: early blastocyst with the trophectoderm (cyan) and inner cell mass (pink). Late blastocyst: the primitive endoderm (yellow) and epiblast (purple). Post-implantation (epiblast): ectoderm (purple); mesoderm (green); endoderm (orange). These are the germ layers that will give rise to all body tissues. ESCs are derived from the ICM from the pre-implantation stage blastocyst. *In vitro*, these cells mimic epiblast differentiation into the germ layers through the formation of embryoid bodies. ICM, inner cell mass; TE, trophectoderm; Epi, epiblast; PE, primitive endoderm; Ecto, ectoderm; Meso, mesoderm; Endo, endoderm; ESC, embryonic stem cell.

A central question in the field of mammalian developmental biology is how a small cluster of identical cells gives rise to different, specialized embryonic cell lineages, properly positioned in the embryonic anatomy. In early mammalian embryo development, fertilization, and zygote formation give rise, after several cleavage rounds, to a multicellular structure known as blastocyst. Before its implantation in the maternal endometrium, the early blastocyst harbors the inner cell mass (ICM), a mass of pluripotent cells that form all the tissues of the embryo. The derivation of embryonic stem cells (ESCs) from the ICM under specific culture conditions allows exploring this pluripotentiality *in vitro*. In the embryo, the ICM continues to undergo a series of cellular, molecular and epigenetic events after implantation, becoming, after gastrulation, a more complex structure consisting of the three primordial germ cell layers (endoderm, mesoderm, and ectoderm) that will form the entire organism. Progressively, the developmental potential of the cells becomes more restricted, until they commit to a specific identity (Bedzhov et al., 2014; Figure 1).

**Figure 2:**
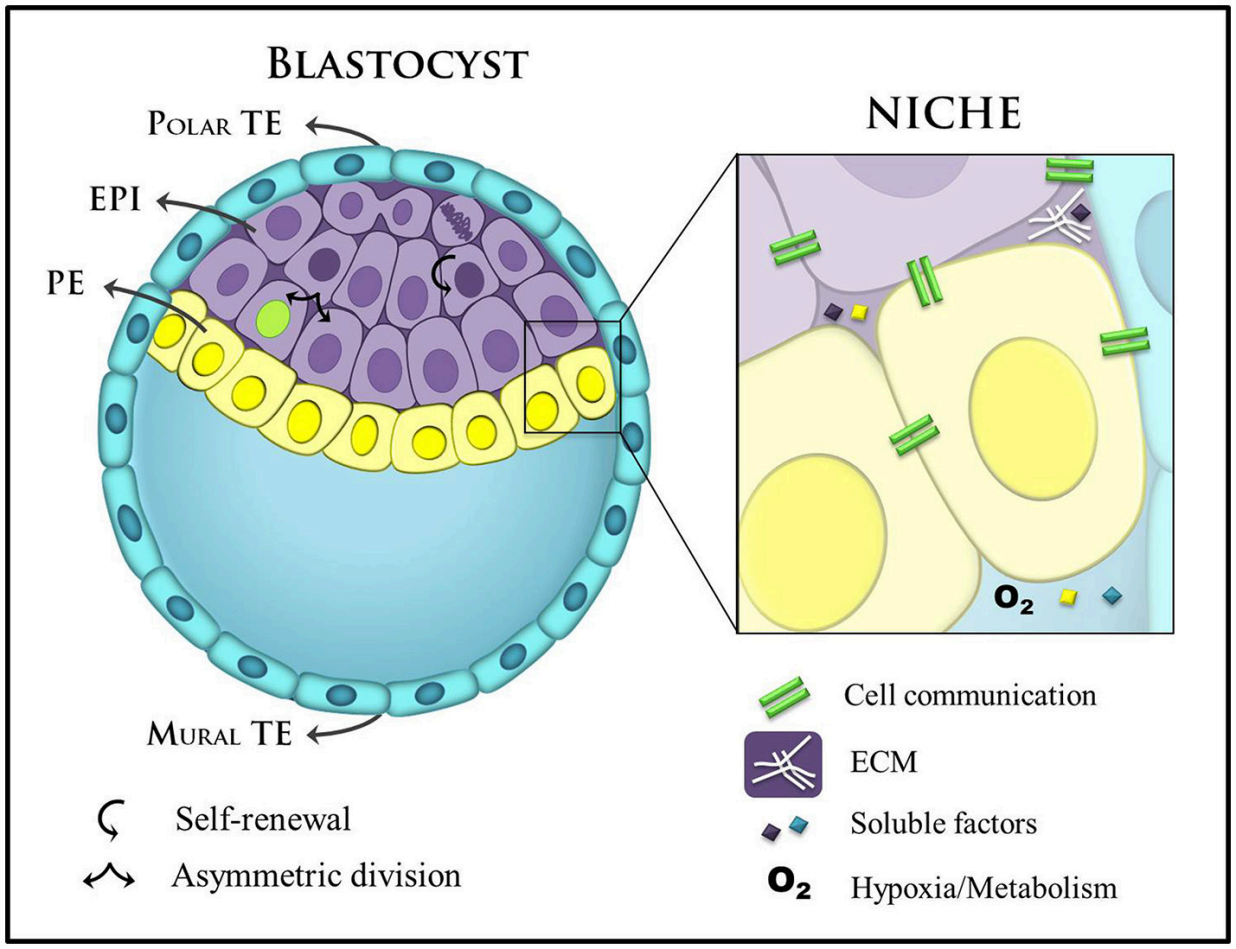
Main components of the blastocyst's niche. A late blastocyst is represented with blastocoel (blastocyst cavity), and the eccentric localization of the ICM containing two distinct layers (EPI: epiblast and PE: primitive endoderm) define the polar and mural trophectoderm (TE). Mural TE initiates uterine implantation. Stem cell niches in the blastocyst are complex and specialized domains and include different cellular components, secreted factors, ECM, and metabolic control. These factors are important players in self-renewal and commitment to cell-fate. ECM, extracellular matrix.

The activation of differentiation programs must be attuned to the cellular microenvironment, which modulates specific transcriptional circuits and gene networks. Thus, the small set of homogeneous cells of the ICM interpret distinct microenvironmental cues, translating them into different cell fate decisions (Figure 2). Amongst these cues, morphogen gradients play a central role in aligning cell fate to embryonic anatomy. Classical experiments showed that certain extracellular ligands can change transcriptomic profile and the ensuing cell fate in a concentration-dependent manner (Gurdon and Bourillot, 2001). Thus, morphogen gradients present in the embryo determine that cells in a homogeneous field will undergo distinct differentiations according to their location in relation to the source (highest concentration) and the sink (lowest concentration) of the morphogen.

A growing number of studies have provided evidence that extracellular vesicles (EVs) can traffic morphogens and miRNAs extracellularly—miRNAs travel between cells and regulate protein expression through post-transcriptional modulation, thereby inducing cell phenotype changes at a non-genomic level (reviewed in Alberti and Cochella, 2017; Gross et al., 2017). Thus, EVs, formerly viewed as cell waste, are now recognized as relevant components in cell communication (Lakkaraju and Rodriguez-Boulan, 2008; Deregibus et al., 2010; Turturici et al., 2014; Benito-Martin et al., 2015; Webber et al., 2015; Pavani et al., 2017). In this review, we discuss the most recent evidence regarding the role of EVs as potential signaling components in both early embryo development and ESCs biology. ESCs are a valuable *in vitro* model to understand events and mechanisms in early embryonic development. Thus, these two experimental paradigms complement each other in their contribution toward our understanding of cell differentiation.

## Cell biology Of extracellular vesicles

The first description of EVs as cell-secreted vesicles was in the 1980s (Trams et al., 1981; Harding et al., 1984; Pan et al., 1985). Since then, they have been referred to by different terms according to their cell/tissue of origin (prostasomes, oncosomes, and apoptotic bodies), size [microparticles, microvesicles (MVs), nanovesicles, and nanoparticles], function (calcifying matrix vesicles, argosomes, and tolerosomes), and presence in the extracellular environment (ectosomes, exosomes, exovesicles, and exosome-like vesicles; Gould and Raposo, 2013; Raposo and Stoorvogel, 2013; Van Niel et al., 2018). As of 2013, all released vesicles are known as extracellular vesicles, and more meticulous isolation and functional analysis are now required to define each type of EV (Witwer et al., 2013). In our literature review on EVs the search included terms such as exosomes, argosome vesicles, nodal vesicular parcels, extracellular lamellar bodies, lamellar vesicles, particles, exovesicles, nanovesicles, and microvesicles. In this review, all of these types of vesicles will be considered as EVs.

**Figure 3:**
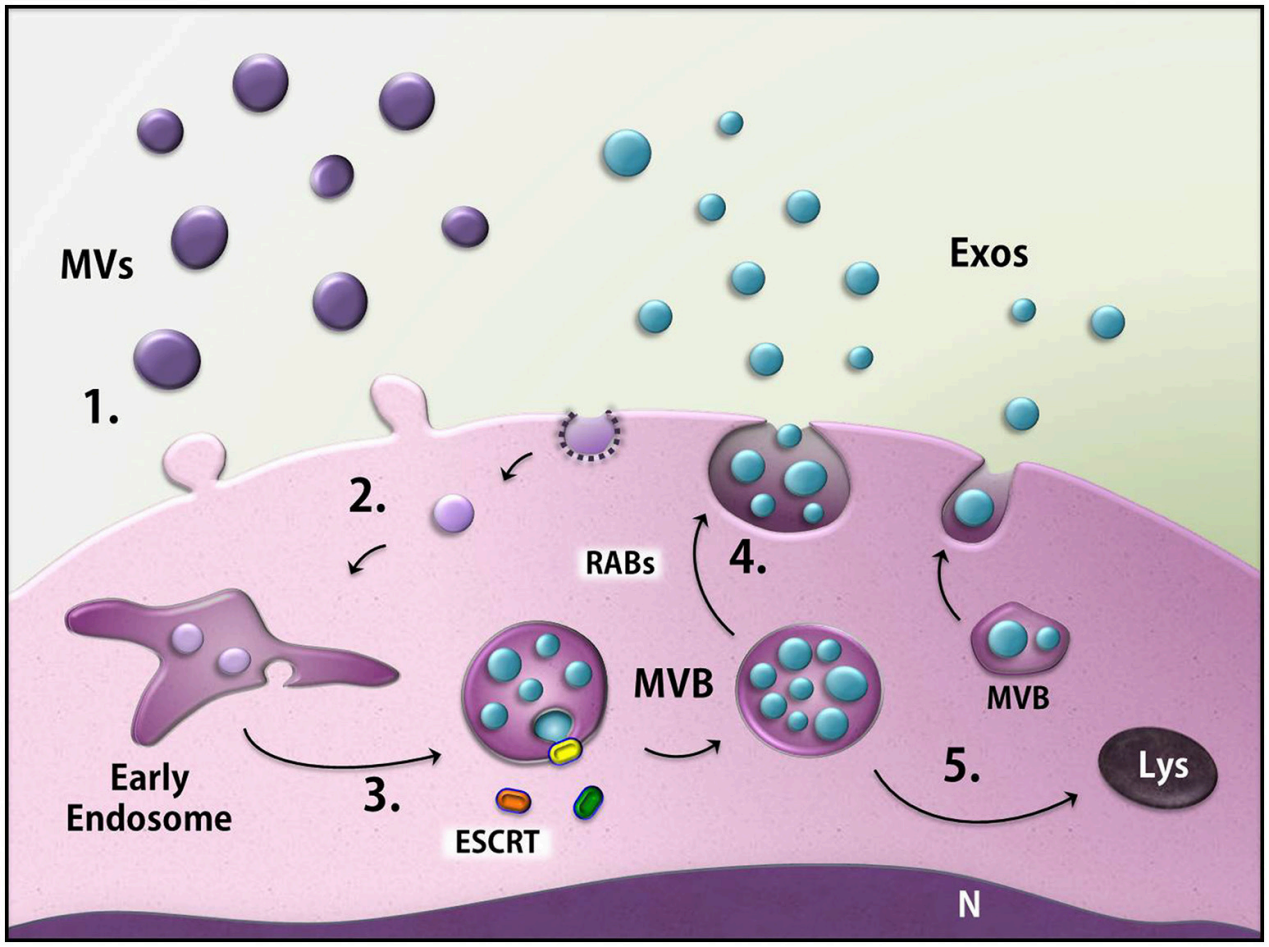
Biogenesis of the extracellular vesicles (EVs). EVs generally consist of microvesicles (purple) derived from the cell membrane (1), as well as exosomes (blue). The latter are found inside multivesicular bodies (MVBs) formed through the endocytic pathway (2) in a process that may involve ESCRT machinery (3). MVBs fuse with the membrane and release the exosomes (4) or can be directed to degradation through the lysosomes (5). MVs, microvesicles; Exos, exosomes; ESCRT, endosomal sorting complex required for transport; MVB, multivesicular body; Rabs, Rab GTPases; N, nucleus, Lys, lysosome.

To date, there is no specific marker for each type of EV, although some tetraspanins (CD9, CD63, and CD81) and members of the ESCRT machinery (ALIX, Tsg101) have been reported to be enriched in exosomes (Kowal et al., 2016). One of the reasons behind the difficulty in finding a common marker lies in the complexity of EV function. The sorted cargo carried by EVs from the cell of origin may exert a specific function on the recipient cell (Nair et al., 2014; Kanada et al., 2015). The cell biology of EV delivery also varies: EV cargo may be delivered by direct fusion between their membrane and the recipient membrane or by endocytosis in the recipient cell (Mulcahy et al., 2014; Lo Cicero et al., 2015; Figure 3). EVs that follow each of these two paths have distinct membrane compositions.

The most commonly described EVs are the so-called exosomes and microvesicles (MVs), which were classified according to their biogenesis and size. MVs (also termed shedding vesicles, microparticles, or ectosomes) are generated from the budding of the plasma membrane (PM), and thus have membrane and content identical to the PM and cytosol, respectively, of their cell of origin. Although MVs are referred to present 100–1,000 nm in diameter, they might not display a size restriction due to their release directly from the PM and can, therefore, overlap the size of exosomes (Lee et al., 2012; Raposo and Stoorvogel, 2013).

Exosomes have a more complex biogenesis than MVs: the inward budding of the endosomal membrane gives rise to intraluminal vesicles (ILVs), resulting in a membrane-delimited compartment known as the multivesicular body (MVB), inside which the ILVs are grouped together. MVBs can either be degraded in lysosomes or fuse with the plasma membrane, thereby releasing the ILVs to the extracellular space. At this step, the ILVs are called exosomes. The nature of exosomes as ILVs limits their size to 30–100 nm in diameter, although isolated exosomes may present a larger size (up to 150 nm) under electron microscopy processing or other techniques analysis (Colombo et al., 2014). The sorting of specific content and the formation and budding of ILVs can either be orchestrated by the endosomal sorting complex required for transport (ESCRT) machinery (ESCRT-dependent mechanism) or by alternative pathways (ESCRT-independent mechanisms). Moreover, some members of the Rab family of GTPases drive the release of MVBs (Ostrowski et al., 2010; Figure 3). However, more studies are necessary to completely unravel the mechanism of exosome biogenesis.

**Figure 4:**
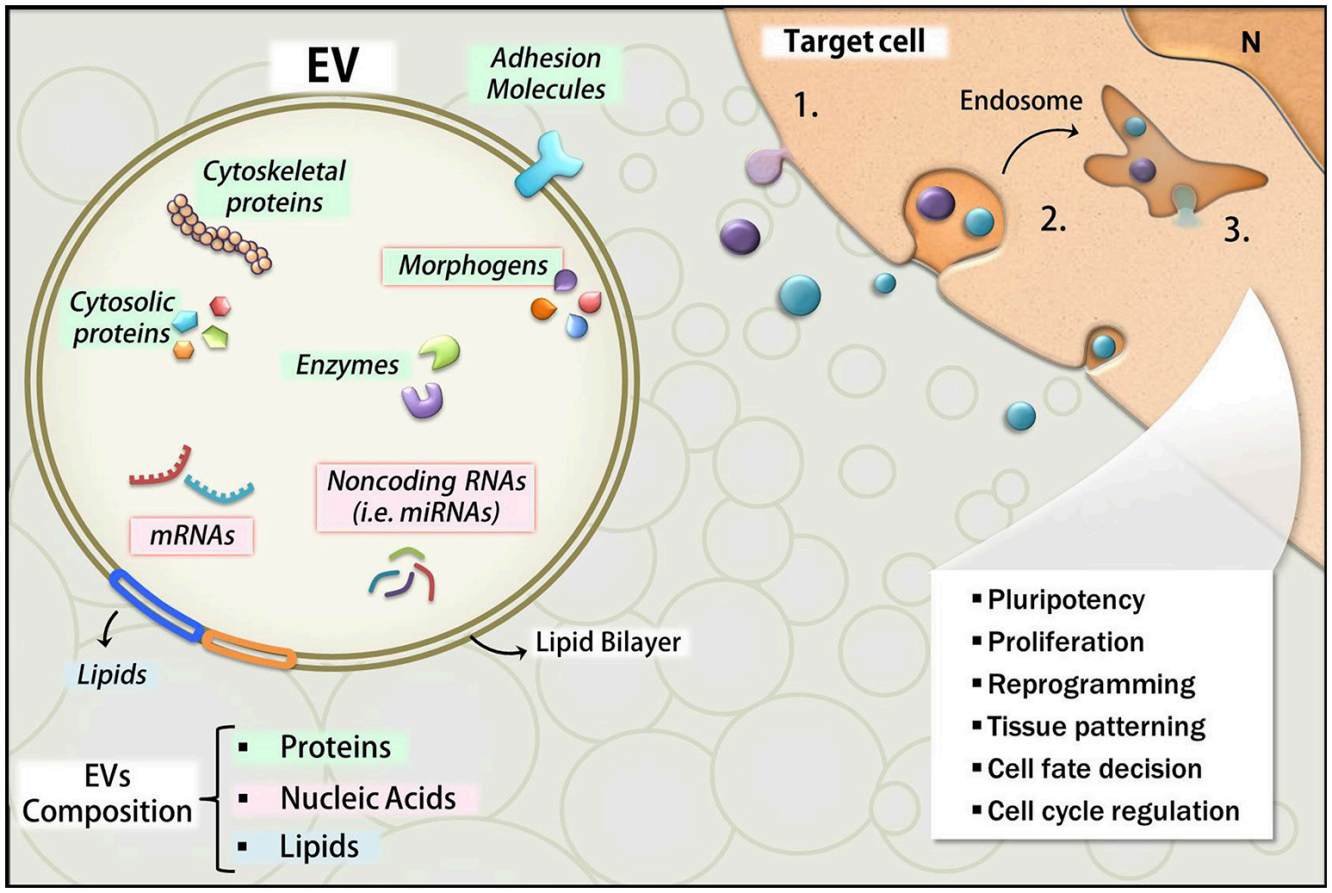
Overview of EVs composition and effects. The main classes of molecules carried by EVs include proteins (green), lipids (blue), and nucleic acids (pink). Many of these molecules are bioactive components and exert functions in the target cells upon their release. RNAs and morphogens are highlighted with red borders. The uptake mechanisms in the target cells include fusion of EVs with the plasma membrane with direct EV content delivery into the cytosol (1), or endocytosis (2), which also leads to EVs content release into the intracellular compartment (3). The developmental effects affected by these events are listed in the illustration.

The literature reports that EVs deliver a broad spectrum of bioactive molecules, including a variety of proteins, lipids, and nucleic acids such as mRNAs and small non-coding RNA (i.e., miRNAs; Choi et al., 2013; Janas et al., 2015; Figure 4). Thus, we will also review here the main categories of EV cargo like miRNA and morphogens known for their importance during development.

## Evidence of EVs as messengers during development

### Cell interactions in the early embryo development: cell membrane extensions guiding signal transduction and EV trafficking

Cell membrane protrusions acting as intercellular bridges have been observed throughout embryogenesis and have been reported in different developmental models and contexts (Fairchild and Barna, 2014; Sagar et al., 2015). These extensions were detected in the blastocoel of early sea urchin and *Xenopus* embryos, in zebrafish and sea urchin gastrulation, in mouse and chick limb bud development, in mouse and zebrafish neural tube development, and in *Drosophila* wing imaginal disc (referred to as cytonemes; Miller et al., 1995; Ramírez-Weber and Kornberg, 1999; Cho et al., 2000; Wood and Martin, 2002; Salas-Vidal and Lomeli, 2004; Gradilla and Guerrero, 2013; Sanders et al., 2013; Rojas-Ríos and González-Reyes, 2014). Many of these studies demonstrated trafficking of morphogen signaling components along cell membrane protrusions, including ligands and/or receptors involved in Notch, Decapentaplegic (Dpp), Sonic Hedgehog (SHH), FGF, EGF, BMP, or Wnt signaling pathways. This form of cell communication mechanism has been extensively reviewed lately (Heckman and Plummer, 2013; McMahon and Hasso, 2013; Fairchild and Barna, 2014; Gradilla et al., 2014; Kornberg, 2014; Rao Damerla et al., 2014; Stanganello et al., 2015; Wood and Rosenbaum, 2015). The most intriguing finding is that vesicles can move along cell extensions. Such vesicles are shed from the membrane and referred as ectosomes. Thus, projections can either be a source of vesicles (ectosomes) or act as a guiding path for vesicle delivery.

During the compaction in the 8-cell stage embryo, cell-cell interactions involving long and thin actin-based cellular projections named filopodia are key mediators of morphological changes. According to this model, cells use filopodia to increase their attachment to neighboring cells in an E-cadherin-dependent manner, thereby controlling changes in cell shape that lead to compaction (Fierro-González et al., 2013). This event results in the formation of an exterior blastomere layer (trophectoderm, TE) lining the innermost cells (ICM). Trophectodermal cells then begin pumping the environmental fluids responsible for generating the blastocyst cavity (the blastocoel). The blastocyst becomes spherical, with the ICM cells positioned at one pole and TE cells surrounding the structure (Figure 1). The region where TE cells remain in contact with the ICM is the polar TE, whereas the TE cells separated from the ICM by the blastocoel correspond to mural TE (Figure 2). Recently, a study provided evidence for intercellular communication between TE and ICM via EVs, which move along filopodia that extend across the blastocoel, in the mouse blastocyst, linking the mural TE to the ICM (Salas-Vidal and Lomeli, 2004). Although there is no direct evidence yet that these vesicles are secreted by ICM, trophoblast cells do respond to EVs. A recent work showed that laminin and fibronectin present in EVs of ICM derived ESCs interacted with integrins of the TE cells surface which induced the JNK and FAK signaling cascades and also TE cells migration. When injected into the blastocoel of mouse embryos, the ESCs EVs were able to increase the blastocysts implantation efficiency (Desrochers et al., 2016). The importance of EVs in early development is also suggested in other mammals. Human extravillous trophoblasts obtained from early placenta release immunomodulatory EVs in culture (Kshirsagar et al., 2012). Similarly, porcine endometrium and choriollantoic membrane secrete EVs and a porcine trophectoderm cell line releases miRNA-containing EVs that stimulate endothelial cell proliferation (Bidarimath et al., 2017). Together, these results underscore the importance of EV-mediate cell communication not only in the development of the early embryo but also in the establishment of the embryonic-maternal interface.

Cilia can also mediate cell-cell communication (reviewed in Wang and Barr, 2016). For example, the gastrulating embryo's node harbors nodal pit cells, whose rotating cilia create an unidirectional fluid flow that is essential for determination of the left-right embryonic axis (Hirokawa et al., 2009). This fluid flow carries EVs that contain Shh and retinoic acid to the left side of the embryo, triggering their respective signaling pathways unilaterally (Tanaka et al., 2005). Likewise, EVs enriched with the stem cell marker prominin-1 (CD133) were observed at the tip of primary cilia in the mouse neuroepithelium specifically at the onset of neurogenesis, suggesting that they may be linked to the state of differentiation of the neural progenitor cells. The authors also suggested that EVs budding from the cilia tip may be a possible mechanism to control cilia length (Dubreuil et al., 2007; Wood and Rosenbaum, 2015).

Altogether, these findings point toward an orchestrated mode of intercellular communication, in which cell membrane projections may act as a source of EVs or guide the direction of EVs trafficking in a particular microenvironment.

### EVs in morphogen delivery

To ensure dynamic and accurate cell positioning throughout the process of embryo patterning and growth, cells convey positional information based on the perception of mobile signaling molecules, such as morphogens. Morphogens can be ligands that bind to cell-surface receptors, leading to targeted gene expression. They are secreted by a group of cells and act at long distance in a concentration-dependent manner, resulting in a combined regulation of specific gene expression that confers a particular cell identity (reviewed by Ashe and Briscoe, 2006).

Some morphogens (Wnt and Hh) undergo lipid modifications (palmitoylation or cholesterol modification) that are essential for their signaling capabilities, but also impair their free diffusion in the extracellular milieu. Thus, the long-range effects of the lipid-modified morphogens require transport mechanisms from their production sites to their targets (Panáková et al., 2005; Eaton, 2006). Besides multimer formation and lipoprotein association, one possible mechanism to control the trafficking of lipid-modified morphogens involves their packaging and anchoring to membranous extracellular vesicles (Rogers and Schier, 2011; Akiyama and Gibson, 2015; Teimouri and Kolomeisky, 2015).

#### Evidence of morphogens and EVs in development

##### Hedgehog

The hedgehog pathway was first identified 38 years ago in fly genetic screens as a relevant part of embryonic axial segmentation. Since then, the Hh pathway has been established as an important component of embryonic, stem cell and cancer biology. Its intercellular signaling has been extensively reviewed elsewhere (Kugler et al., 2015; Wu et al., 2017). In brief, it is an evolutionarily conserved pathway whose core components are the secreted ligand Hh, the transmembrane receptor Patched 1, the intracellular G-protein Smoothened and transcription factors GLI1-3. In vertebrates, three HH orthologs have been identified: Sonic (Shh), Indian (Ihh), and Desert Hedgehog (Dhh).

In developing mouse embryos, left-right axis determination results from the leftward movement of fluid at the ventral node (nodal flow), which triggers a left-right gradient of morphogens and signaling that will convey asymmetric development to internal organs. Tanaka et al. (2005) showed that FGF signaling induces secretion of the EVs containing Sonic Hedgehog (Shh) and retinoic acid at the node of the gastrula's primitive streak. Furthermore, the authors provided evidence suggesting that the fluid flow at the node promotes leftward movement of EVs. Asymmetric accumulation of EVs specifically activates the non-canonical hedgehog signaling pathway in the left side of the embryo. Thus, the ultimate consequence of this asymmetric EV transport is differential changes in intracellular calcium levels and gene expression (Tanaka et al., 2005).

Another study showed that primary notochord cells derived from early chick embryos release Shh in two distinct EV pools (the first fraction obtained from 150,000 g ultracentrifugation (P150), and another fraction obtained from (P150) subjected to 450,000 g ultracentrifugation (P450). These two EV populations presented different protein and miRNA composition, leading to different abilities in activating endogenous target genes. For instance, only the P150 fraction, which specifically contained ß1-integrin, could activate HNF3b, Olig2, and Nkx6.2 expression during the differentiation of mouse embryonic stem cells (mESCs) to ventral neuronal progenitors. Overall, the study provided evidence that distinct classes of EVs carry different signal-modulatory accessory molecules that could modify the effect of Shh in target cells (Vyas et al., 2014).

Hh trafficking and secretion depends on the ESCRT system. In fly embryos, Hh and ESCRT components are secreted together into the extracellular space and can be observed at the surface of the recipient cells (Matusek et al., 2014). Downregulation of ESCRT members leads to retention of Hh in the apical region of the cells in fly wing imaginal discs and consequently, defects in Hh-mediated signaling.

Targeting of Hh prior to secretion is an important prerequisite for proper signaling. The manner in which Hh ligands are secreted is not entirely understood. Molecular mechanisms that regulate polarized targeting of proteins to the apical or basolateral membranes are critical for Hh secretion, but additional processing pathways seem to exist. In the *Caenorhabditis elegans* epidermis, secretion of Hh-related peptides is mediated by their incorporation into MVB intraluminal vesicles, which could be subsequently released as EVs in the apical membrane (Liégeois et al., 2006). This process requires a subunit of the vacuolar H^+^-ATPase (V-ATPase), independent of its role in proton pump activity (Liégeois et al., 2006).

Hh- and Shh-containing EVs can also reach target cells by traveling across cytonemes, specialized signaling filopodia found both in the *Drosophila* wing and in the chick limb bud (Ramírez-Weber and Kornberg, 1999; Sanders et al., 2013). This movement along cytonemes is an alternative dispersion route that forms an oriented gradient of Hh (Bischoff et al., 2013; Sanders et al., 2013; Gradilla et al., 2014). Also related to cell membrane extensions, another study showed that human ESCs (hESCs) contain Shh signaling machinery in their primary cilia that may be involved in the regulation of hESC differentiation and/or in the maintenance of the undifferentiated state. Although the authors observed the presence of structures similar to EVs adherent to the surface of hESCs, they did not confirm experimentally the transport of Shh through EVs (Kiprilov et al., 2008).

##### WNT

The Wnt signaling pathways govern essential biological processes during embryonic development, including stem cell renewal and differentiation, cell proliferation, cell polarity and migration, organogenesis, and tissue patterning, among others (reviewed by Loh et al., 2016). Extracellular Wnt proteins bind to Frizzled receptors and stimulate intracellular signaling cascades that include the canonical or Wnt/β-catenin dependent pathway and the non-canonical or β-catenin-independent pathway. The latter can be divided into the Planar Cell Polarity pathway and the Wnt/Ca^2+^ pathway (Komiya and Habas, 2008; MacDonald et al., 2009).

Wnt secretion through EVs was suggested as a model to explain the *Drosophila* Wnt 1 (Dwnt-1 or Wingless, Wg) extracellular trafficking carried by argosomes in the *Drosophila* wing imaginal disc (Greco et al., 2001). However, it was later discovered that argosomes were lipoprotein particles, rather than membranous vesicles (Greco et al., 2001;). Later, it was shown that Wg is secreted on vesicles in fly larval imaginal discs and neuromuscular junctions (Korkut et al., 2009; Gross et al., 2012; Koles et al., 2012). In the latter study, Wg secretion was associated with the Wg transmembrane binding protein Evenness Interrupted/Wntless/Sprinter (Evi/Wls/Srt) and Wg was demonstrated to be transported across larval neuromuscular junction synapses in Evi-containing EVs (Korkut et al., 2009; Koles et al., 2012). Further, knockdown of Evi in the wing imaginal discs results in intracellular accumulation of Wg (Gross et al., 2012). Human cell lines also secrete active Wnt in EVs that carry Evi/WIs/Wnts (Gross et al., 2012). These data strongly support the idea of Evi-containing EVs as secretory route for extracellular transport of active Wg. However, Evi does not seem to be required for exosome production in fly cell lines. Also, Evi and Wg colocalize only near the expression domain but not in the extracellular milieu of the wing imaginal disc (Beckett et al., 2013). Together, these data suggest that Evi might mediate Wg trafficking to the membrane prior to secretion but that Evi-containing exosomes do not contribute to the establishment of extracellular Wg gradient.

Regarding vertebrate embryos, chick dermomyotomal cells emit filopodia toward the overlying epithelia. Although the release of EVs has not been detected, these filopodia contain motile punctae that retrogradely transport the Frizzled-7 Wnt receptor, suggesting that vesicular transport might play a role in long-distance Wnt signaling (Sagar et al., 2015).

These evidence indicate the value of further exploring the role of EVs in Wnt signaling, using experimental methods such as visualization, validation, and content and functional analysis of EVs.

##### NOTCH

The Notch signaling pathway controls many important developmental events, such as cell differentiation decision, polarity, and patterning (reviewed by Mašek and Andersson, 2017). Notch is a transmembrane protein whose intracellular domain is cleaved upon cell contact after binding to Delta. Increase in the levels of the Notch intracellular domain elicits its downstream effects. Some studies first speculated that the presence of Delta in EVs could be a mechanism for cell contact-independent activation of Notch signaling (Le Borgne and Schweisguth, 2003; Kopan and Ilagan, 2009). This hypothesis was validated by detecting Delta-like 4 (Dll4), a member of the Delta-like ligands that is essential for angiogenesis and vascular development, in EVs released by human endothelial cells. EVs containing Dll4 were either inserted in the plasma membrane or internalized into target cells and increased angiogenesis by inhibiting Notch signaling. However, the mechanism through which vesicular Dll4 secretion inhibits the Notch pathway, instead of activating it, remains to be elucidated (Sheldon et al., 2010). Strikingly, Dll4, similar to Wnt10b, colocalizes with EV biogenesis markers and is released by EVs derived from mESCs, suggesting a role in stem cell biology (Cruz et al., 2017).

##### BMPs

Bone morphogenetic proteins (BMPs) are members of the transforming growth factor-beta superfamily (TGFb) that act as morphogens regulating tissue patterning, body axes organization, growth, and maintenance of stem cell niches during embryonic development of different species. BMPs interact with their specific receptors (BMRPs) on the cell surface by triggering the mobilization/phosphorylation of SMAD family members that translocate into the nucleus and regulate gene expression (reviewed by Wang et al., 2014; Bier and De Robertis, 2015). BMP morphogen gradients have been extensively studied in a variety of animal models, but only recently BMP2/4 was identified in EVs (Draening et al., 2018). That report demonstrated that EVs isolated from zebrafish embryos contained biologically active BMP2/4, which activated BMP-dependent gene expression in cells. Inhibition of EV secretion reduced SMAD1/5/9 phosphorylation and downstream transcription activity, generating morphological phenotypes reminiscent of BMP inhibition. These findings suggest that EV can play a role in the establishment of BMP morphogen gradient during zebrafish development (Draening et al., 2018).

### EVs in miRNA delivery

#### Role of miRNAs in development and ESC biology

Small regulatory non-coding RNAs support morphogen gradients at the post-transcriptional level and contribute toward spatiotemporal accuracy of developmental gene expression programs (Levine et al., 2007; reviewed in Benkovics and Timmermans, 2014). Among this group of RNA molecules, microRNAs (miRNAs) emerge as important modulators of embryonic development due to their capacity to directly downregulate target mRNA translation.

miRNAs are small and single-stranded non-coding RNAs with 22–24 nucleotides, many of which are highly conserved among different organisms. They were first discovered in *C. elegans* and later found in most other eukaryotes, including plants and humans (Lee, 1993; Lee et al., 2007; Carlsbecker et al., 2010; Meunier et al., 2013). Mature miRNAs bind to specific target sequences in the non-coding regions of mRNAs. This interaction leads to mRNA degradation or translational repression (reviewed in Iwakawa and Tomari, 2015), and thus play a crucial role in regulating gene expression in several biological processes, including early embryo development and stem cell differentiation (Kloosterman and Plasterk, 2006; Tripathi et al., 2011; Berardi et al., 2012; Ivey and Srivastava, 2015).

Some miRNAs (i.e., miR-290 cluster) that specifically are expressed in the embryo with a dynamic expression pattern during development are also expressed in the ESCs (Houbaviy et al., 2003; Aboobaker et al., 2005; Medeiros et al., 2011; Asikainen et al., 2015). Removal of DICER, an enzyme involved in the biosynthesis of miRNAs, is lethal to early mouse embryos (Bernstein et al., 2003). Strikingly, disruption of DICER impairs differentiation and proliferation of mouse ESCs (Kanellopoulou et al., 2005; Murchison et al., 2005; Wang et al., 2007). Several other studies have identified the mechanisms by which particular miRNAs contribute to cell fate choices, transition between pluripotency and differentiation, and developmental patterning (Marson et al., 2008; Spruce et al., 2010; Pernaute et al., 2011; Garg et al., 2013). This topic has been extensively reviewed elsewhere (Bartel, 2018); here, we highlight the paracrine role of miRNAs released through EVs.

#### Evidence of miRNAs and EVs in development and ESCs biology

A growing body of evidence demonstrates that miRNAs are secreted from cells in a selective manner (Kosaka et al., 2010, 2013; Pigati et al., 2010; Cheng et al., 2014). Furthermore, extracellular miRNAs have been differentially detected in physiological and pathological conditions, indicating that these molecules may serve as biomarkers to monitor disease states (Heishima et al., 2015; Li et al., 2015; Hu et al., 2016; Faccini et al., 2017). Several reports have shown that extracellular miRNAs are sorted, secreted and transported inside EVs Valadi et al., 2007; Squadrito et al., 2014; Cha et al., 2015; van Balkom et al., 2015; Zhang et al., 2015. They were also found to be associated with lipoproteins (HDL/LDL; Vickers et al., 2011; Rayner and Hennessy, 2013) and RNA-binding proteins, such as Argonaute 1 and 2 (Ago1 and Ago2; Arroyo et al., 2011; Turchinovich et al., 2011; Turchinovich and Burwinkel, 2012). Once in the extracellular space, miRNAs are delivered to a target cell, where they interfere with gene expression through the above-mentioned mechanisms.

ESCs have been described as a relevant source of EVs that convey cues for the modulation of stem cell plasticity by supporting cell self-renewal, proliferation, and differentiation (Ratajczak et al., 2006; Yuan et al., 2009; Quesenberry and Aliotta, 2011; Katsman et al., 2012; Quesenberry et al., 2012; Jeong et al., 2014; Khan et al., 2015). The first example supporting this idea came from Ratajczak and coworkers. Their work showed that mESCs-derived EVs induced epigenetic changes, through unclear mechanisms, in hematopoietic progenitors by transferring mRNA and proteins involved in the up-regulation of early pluripotent and early hematopoietic markers and signal transduction stimulation (Ratajczak et al., 2006). Several reports show that ESCs release biologically active miRNA through EVs. When mESC-derived EVs were incubated together with mouse embryonic fibroblasts (MEFs), the levels of a specific subset of miRNAs increased in MEFs. This subset of miRNAs mostly included miRNAs that were abundantly present in ESCs and were not endogenously expressed in the MEFs (Yuan et al., 2009). The selective transfer of ESCs mRNA and miRNAs via EVs has also been described in cultured retinal progenitor Müller cells, which upregulated pluripotency and early retinal genes and downregulated differentiation genes, together with morphological and miRNA transcriptome changes toward a dedifferentiated progenitor phenotype (Katsman et al., 2012). Recently, mouse retinal progenitor cells (mRPC) were shown to secrete EVs carrying miRNAs (miR-9, miR-182, miR-204, and Let7d), mRNAs (Pax6, Hes1, Sox2, Ki67, Nestin, and GFAP) and proteins (retinogenic growth factors and morphogenic proteins) associated with multipotency and retinal development. Moreover, mRPC-EVs could transfer GFP mRNA and Cre+, activating loxP GFP expression in the recipient cells (by using the Cre-loxP recombination system) which supports a novel intercellular communication mechanism mediated by mRPC EVs during retinal development (Zhou et al., 2018). Another report showed that EVs found in rodent and human embryonic cerebrospinal fluid (eCSF) carry miRNAs and proteins that modulate the IGF-mTORC1 signaling pathway. Similarly, mouse embryonic neural stem cell (eNSC) cultures responded to CSF-derived EVs by activating the IGF pathway and increasing the number of proliferating cells (Feliciano et al., 2014). These data suggest that eCSF-derived EVs may provide extrinsic signals to regulate NSCs proliferation and self-renewal during embryogenesis. In support of this, another study showed that neonatal NSCs from the subventricular zone (SVZ) released *in vitro* EVs containing miRNAs (i.e., miR-9, Let-7, miR-26, and miR-181). When injected *in vivo*, these exosomes were selectively uptaken by microglia and upregulated cytokine secretion. In turn, the conditioned media of the EV-treated microglia reduced NSC proliferation *in vivo*. Thus, NSC proliferation could be modulated by a negative feedback loop whereby NSC-secreted EVs acted on ventricular microglia, which responded by increasing cytokine production that regulated NSC proliferation (Morton et al., 2018).

Another line of evidence has identified a prominent role for EV-mediated signaling in *in vitro* embryonic development. In controlled conditions and in the absence of maternal elements, embryos cultured in groups have improved developmental rates and embryo quality compared to those cultured individually (Lane and Gardner, 1992; Stokes et al., 2005; Ebner et al., 2010; Saadeldin et al., 2014). This suggests that embryos can create their own niche *in vitro*, by releasing intercellular communication factors that enhance survival (Paria and Dey, 1990; Wydooghe et al., 2015). In this context, a variety of cultured embryos (porcine, bovine, human, rabbit) secrete particular miRNAs whose presence has been consistently associated with embryonic robustness, and could be used as biomarkers for embryonic quality (Berardi et al., 2012; Goossens et al., 2013; Maraghechi et al., 2013; Kropp et al., 2014; Rosenbluth et al., 2014; Krawczynski et al., 2015; Liu et al., 2015).

The first report that demonstrated the involvement of EVs in inter-embryonic communication *in vitro* was from parthenogenetic embryos (Saadeldin et al., 2014). Saadeldin et al. (2014) showed that EVs from parthenogenetic embryos contain mRNA of pluripotency genes and can improve the developmental competence of cloned embryos. A recent study showed that when murine embryos were co-cultured with EVs derived from human endometrial MSCs (EV-endMSCs), there was an increase in the blastocyst's total cell number, together with an increase in embryonic secretion of VEGF and PDGF. These results suggest a role for EV-endMSCs in supporting embryo development (Blázquez et al., 2018). Although these studies did not clearly demonstrate the association between miRNAs and EVs (Kropp et al., 2014), we cannot disregard the possibility that miRNA-containing EVs exist in the embryonic culture media. Indeed, this was recently explored in a study demonstrating the role of EVs derived from uterine luminal fluid in embryonic development. Embryos co-cultured with EVs isolated from pregnant mouse uterine fluid (ULF-EVs), increased their expression of miR-21. This increase was less in the presence of an endocytosis inhibitor, suggesting that ULF-EVs transferred their miRNA content to embryonic cells. Further, co-culture with ULF-EVs increased the rate of blastocyst formation and reduced apoptosis (Lv et al., 2018). Altogether, it is pertinent to consider the use of engineered EVs carrying specific miRNAs to optimize *in vitro* embryonic development.

## Perspectives

The data reviewed above highlight the potential role of EVs as a mode of cellular communication during early phases of embryonic development. Release of EVs mediate paracrine cellular communication and induce cell self-renewal and differentiation. Recent findings suggest that EVs act as vehicles for miRNA and morphogens, molecules classically related to phenotype commitment and tissue patterning in the embryonic microenvironment. Although this review advances the role of EVs in embryonic cell signaling as pivotal, several issues remain to be better elucidated. How are EVs used for cell fate decision in the ICM? Are EVs important in defining transcriptional signatures in the early embryo? Are EVs playing a role as autocrine signaling components as well? Do EV cargoes refine morphogen signals in tissue patterning in later developmental stages? It is clear that EVs have just started to receive special attention in developmental biology.

## Author contributions

LC: Conceived, writing and figures preparation; JR: Writing; RI: Writing; ML: Conceived and writing.

### Conflict of interest statement

The authors declare that the research was conducted in the absence of any commercial or financial relationships that could be construed as a potential conflict of interest.
